# Artificial vision with wirelessly powered subretinal electronic implant alpha-IMS

**DOI:** 10.1098/rspb.2013.0077

**Published:** 2013-04-22

**Authors:** Katarina Stingl, Karl Ulrich Bartz-Schmidt, Dorothea Besch, Angelika Braun, Anna Bruckmann, Florian Gekeler, Udo Greppmaier, Stephanie Hipp, Gernot Hörtdörfer, Christoph Kernstock, Assen Koitschev, Akos Kusnyerik, Helmut Sachs, Andreas Schatz, Krunoslav T. Stingl, Tobias Peters, Barbara Wilhelm, Eberhart Zrenner

**Affiliations:** 1 Centre for Ophthalmology, University of Tübingen, Schleichstraße 12–16, 72076 Tübingen, Germany; 2 STZ EyeTrial at the Centre for Ophthalmology, University of Tübingen, Schleichstraße 12–16, 72076 Tübingen, Germany; 3 Mobility Trainer, Mozartweg 11, 72076 Tübingen, Germany; 4 Department of Ophthalmology, Semmelweis University, Tomo u. 25–29, 1083 Budapest, Hungary; 5 Clinic Friedrichstadt, Friedrichstraße 41, 01067 Dresden, Germany; 6 MEG Center, University of Tübingen, Otfried-Müller-Straße 47, 72076 Tübingen, Germany; 7 Retina Implant AG, Gerhard-Kindler-Straße 8, 72770 Reutlingen, Germany; 8 Department of Otorhinolaryngology, Division of Paediatric Otorhinolaryngology and Otology, Klinikum Stuttgart, Bismarckstrße 8, 70156 Stuttgart, Germany

**Keywords:** artificial vision, neuroprosthetics, retinitis pigmentosa, electronic implants

## Abstract

This study aims at substituting the essential functions of photoreceptors in patients who are blind owing to untreatable forms of hereditary retinal degenerations. A microelectronic neuroprosthetic device, powered via transdermal inductive transmission, carrying 1500 independent microphotodiode-amplifier-electrode elements on a 9 mm^2^ chip, was subretinally implanted in nine blind patients. Light perception (8/9), light localization (7/9), motion detection (5/9, angular speed up to 35 deg s^−1^), grating acuity measurement (6/9, up to 3.3 cycles per degree) and visual acuity measurement with Landolt C-rings (2/9) up to Snellen visual acuity of 20/546 (corresponding to decimal 0.037 or corresponding to 1.43 logMAR (minimum angle of resolution)) were restored via the subretinal implant. Additionally, the identification, localization and discrimination of objects improved significantly (*n* = 8; *p* < 0.05 for each subtest) in repeated tests over a nine-month period. Three subjects were able to read letters spontaneously and one subject was able to read letters after training in an alternative-force choice test. Five subjects reported implant-mediated visual perceptions in daily life within a field of 15° of visual angle. Control tests were performed each time with the implant's power source switched off. These data show that subretinal implants can restore visual functions that are useful for daily life.

## 1. Introduction

**Figure 1.**
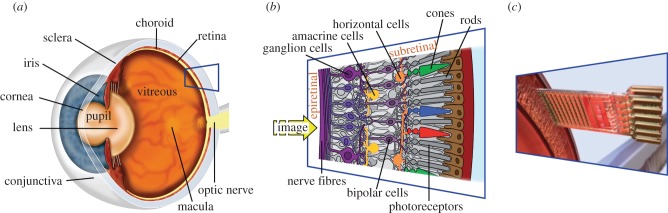
Human eye. (*a*) The structures of the eye and (*b*) the retinal layers in detail. (*c*) The function of photoreceptors lost because of hereditary degeneration can be partially replaced by a subretinal chip. The chip carries a microphotodiode array with amplifiers and electrodes on a 3 mm × 3 mm area and is surgically placed subretinally in the location corresponding to the layer of degenerated photoreceptors.

Photoreceptors—cones and rods—in the outermost layer of the retina convert light into an electrical current that provides input to the second layer, i.e. the bipolar cell neurons (figure 1*a*,*b*). These signals are processed within the retinal neuronal network and are forwarded via the ganglion cell axons that form the optic nerve to the lateral geniculate nucleus and then to the visual cortex.

In most hereditary retinal diseases, such as retinitis pigmentosa, the photoreceptors progressively degenerate, often causing blindness in adult life, and there is no therapy available to treat this disease. However, the remaining visual pathway, from the bipolar cells onwards, remains largely functional. Therefore, various groups have attempted to replace photoreceptive function using technical devices to restore visual sensation in these patients.

Approaches of stacking photodiodes in series to use, similar to solar cells, the light of an image itself for neuronal stimulation have been shown to be feasible *in vitro* [1]. However, complicated goggles with high-luminance laser stimulators are necessary for providing sufficiently bright images to drive such passive light sensors.

**Figure 2.**
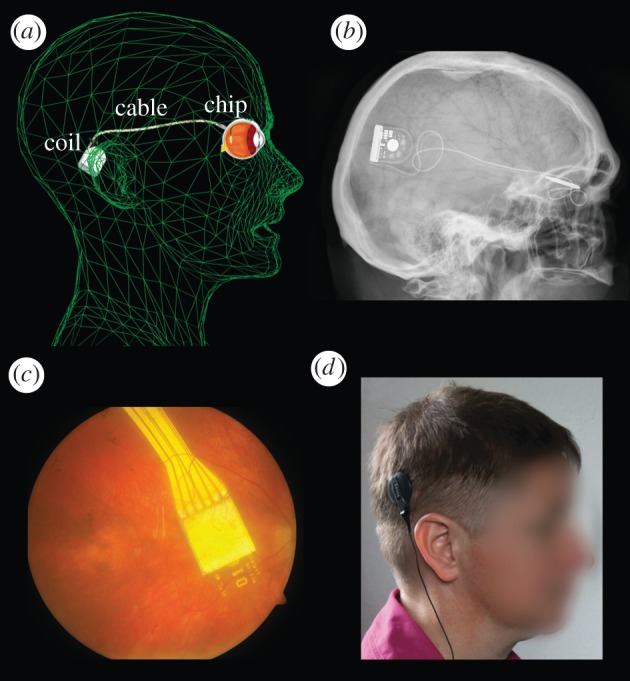
The alpha-IMS subretinal implant. (*a*,*b*) The subdermal coil behind the ear provides power and sends control signals via a subdermal cable and a thin intraocular foil to the chip in the eye. (*c*) The chip is placed surgically beneath the fovea and contains 1500 pixels (independent microphotodiode-amplifier-electrode elements) on a 3 mm × 3 mm area. Via a thin black cable, a small battery pack (not shown) powers the primary external coil, (*d*) which is magnetically kept in place above the subdermal coil behind the ear and provides power and signals via transdermal electric induction.

While some groups favour an epiretinal [2] or suprachoroidal [3,4] approach for stimulating primarily ganglion cells, we aimed to restore visual function in these patients by the means of subretinally implanted microelectronic devices that use light-sensitive detector arrays and amplifiers to convert light into signals that can stimulate the bipolar cell neurons [5] via tiny metal electrodes (figure 1*c*). Following preclinical work [6–10] and a clinical pilot trial using a cable-bound implant (2005–2009) [11,12], we report here the first results from implants equipped with wireless power and signal transmission (figure 2), which allows the patients to use the implant at home or outdoors, providing a diamond-shaped visual field of 15° across chip corners.

## 2. Methods

### (a) Subretinal alpha-IMS visual implant

The implant's core is an active subretinal chip with 1500 pixels (see figure 1 and electronic supplementary material, figures S1 and S2). Each pixel has a photodiode, which is used to analyse the brightness of the incoming light, an amplification circuit and an electrode for charge transfer to the adjacent retinal layers. The chip typically records images five to seven times per second (working frequency of 5–7 Hz, adjustable in a wide range) and provides the bipolar cells with a ‘point-by-point electrical image’ of the luminance distribution, typically consisting of 1 ms pulses with amplitudes that are correlated with the luminance at each point. From the bipolar cells onwards, the signal is processed via the remaining visual pathway. The chip size is approximately 3 mm × 3 mm and is approximately 70 µm thin when placed on polyimide foil (thickness approx. 17 µm), which leaves the subretinal space in the upper temporal periphery through the choroid and the sclera. The foil is connected to the power supply cable, which, after a loop in the orbit, leads to the retroauricularly placed subdermal coil (figure 2). Here, the inductive transfer of energy and control signals from the skin to the implant are provided via an external coil from a battery pack in the handheld control unit. This battery pack (see the electronic supplementary material, figure S3) has two knobs for adjusting the amplification and the gain of the amplifiers, thereby adjusting the overall brightness and contrast of the perception according to the particular luminance conditions. This adjustment is performed by the patient after training and is based on subjective perception.

The biocompatibility results [8–10,13] as well as the surgical implantation procedures [14,15] were previously published.

The chip is positioned beneath the foveal region at a predefined position based on optical coherence tomography, fluorescein angiography and retinal pigment clustering ([16]; figure 2*c*). The implant provides a diamond-shaped visual field of 10° × 10°, diagonally 15°. The implant, called alpha-IMS is manufactured by Retina Implant AG (Reutlingen, Germany) with the electronic chip design provided by the Institute for Microelectronics, Stuttgart (IMS), Germany.

### (b) Patients

Nine patients (four females, five males) aged 46.9 ± 7.2 years (35–62 years) years participated in the first module, which completes the monocentric part of a multicentre trial (www.clinicaltrials.gov, NCT01024803). The patients received the subretinal visual implant in one eye (the one with the worst visual function). Visual function prior to implantation was light perception without correct light source localization (eight patients) or complete blindness (no light perception, one patient) caused by hereditary retinal diseases (eight patients with retinitis pigmentosa, one patient with cone–rod dystrophy). None of the subjects had other eye diseases that might affect the visual pathway. The electronic supplementary material, and table S1 provides more details on the patients' characteristics.

Written informed consent in accordance with the declaration of Helsinki was obtained from all the subjects prior to inclusion in the study.

### (c) Efficacy testing

**Figure 3.**
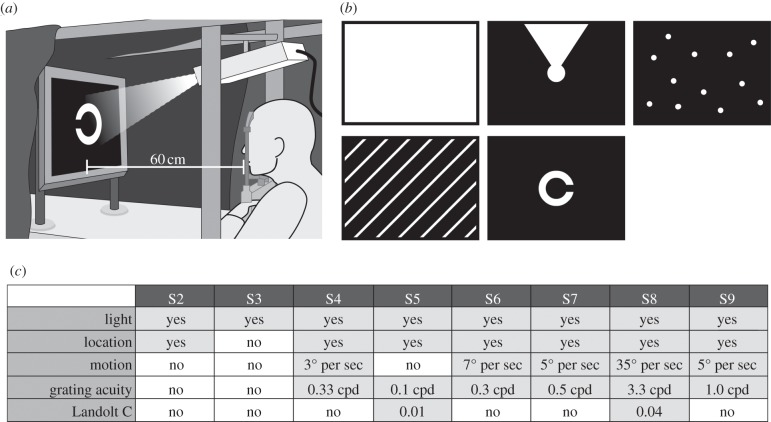
Screen tasks. (*a*) Using a projector-screen set-up*. (*b*) Light perception threshold, light source localization, motion detection, grating acuity (spatial resolution of periodic stripe pattern) and visual acuity (standardized Landolt C-shaped optotypes) were assessed. A four-alternative-forced-choice mode (4AFC) was applied, except for light perception where a 2AFC was used. (*c*) Light perception with the implant was possible in eight subjects, light source localization was possible in seven subjects, motion detection (angular speed up to 35 deg s^−1^) was possible in five subjects, grating acuity measurement (up to 3.3 cpd) was possible in six subjects, and visual acuity measurement with Landolt C-rings was possible in two subjects (20/2000 and 20/546, corresponding to gap sizes of 1.6° and 0.45° visual angle). At least 75% (in 2AFC) or 62.5% (in 4AFC) correct responses were required to pass the test (‘yes’). *Grating acuity measurement and Landolt C-ring tests were carried out on a table with subject S5 using a set-up similar to that shown in figure 4.

**Figure 4.**
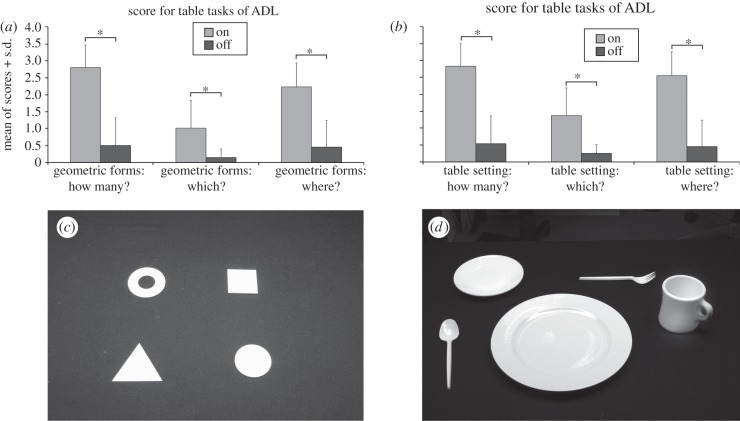
Table tasks of ADL. First, four geometrical objects (out of six possible objects: circle, ring, crescent, triangle, square, rectangle) were placed on the table. The patient was not aware of the maximal possible number of the shapes put in front of him. The patient was asked to report, how many, where and which shapes he/she could see. For every question, the number of correctly identified, discriminated and localized objects was documented on a scale ranging from 0 to 4. In the second part of the test, a table setting was presented using white tableware. A large white plate in the middle was obligatory and known to the patient. Around the plate, four objects (out of six possible objects: middle-sized plate, small plate, cup, spoon, fork or knife) were arranged. Again, recognition, description and localization were reported and documented. (*a*,*b*) Significant differences were found between the ON/OFF (grey bars and black bars, respectively) implant power supply conditions for all tasks performed on a table with geometrical shapes (*c*) and (*d*) tableware objects in all eight patients. Whiskers indicate the standard deviation. The significance level was reached for all six questions (the asterisk indicates *p* < 0.05).

As specified by the study protocol, the following efficacy tests were performed: (i) standardized screen tasks [17,18], including tests of visual acuity (figure 3*a,b*); (ii) table tasks of activities of daily living (ADL; figure 4*c,d*); (iii) letter recognition; and (iv) reports of the experience with the visual implant in daily life. As a control, all the tests except patient reported use in daily life were administered in two conditions, implant power source ‘ON’ and ‘OFF’, in a randomized order, and the subjects were masked to the condition.

The other eye was always occluded during the tests. Distance-corrected refraction was provided for the study eye by means of regular spectacles. The tasks were performed repeatedly during up to 18 visits in the nine-month period. A detailed description and explanation of the testing procedures are the subjects of a recent publication [19].

#### (i) Standardized screen tasks

For measurements of the very basic visual functions, the basic assessment of light and motion test was applied [17]. The subtests of the test battery are designed as two- or four-alternative-forced-choice tests measuring (i) light perception threshold in full field illumination, (ii) light source localization and (iii) motion detection with a moving random dot pattern (figure 3*b*).

The standardized basic grating acuity (BaGA) test [20] was used for grating acuity, also in a two- or four-alternative-forced-choice mode (at least 12 trials in each test), where a black-and-white striped pattern was presented at spatial frequencies of 0.1, 0.33, 1.0 and 3.3 cycles per degree (cpd), and the patient was asked to report on the grid orientation. If a patient passed some, but not all, of the predefined spatial frequencies, we manually applied striped patterns of 0.4, 0.5 cpd, etc., from a PowerPoint-based presentation. A ratio of 1 : 5 of the thickness of the white : black stripes was used, as this ratio was generally the most acceptable for the patients.

Subsequently, visual acuity with Landolt C-rings was tested by an automated Freiburg acuity and contrast test [18] or a manual PowerPoint-based presentation of 12 contrast-reversed, standardized Landolt C-rings for each gap size in four possible gap directions.

Subject S5 had difficulties if the grating pattern and Landolt C-rings were presented on the screen. Therefore, for this subject, we used a paper-grating pattern and paper Landolt C-rings in reverse contrast on a table (black background). The spatial and visual resolutions were calculated for the corresponding eye distance.

The dynamic range of the chip's sensitivity to saturation spans is approximately two log units and is adjustable within a luminance range of 1 to 100.000 cd m^−2^. The luminance range provided by the projector on the screen was adjustable by means of neutral density filters in steps of 0.15 log units and ranged between 200 and 2000 cd m^−2^ on the white target and 0.5–20 cd m^−2^ on the background.

#### (ii) Table tasks of activities of daily living

We used a standardized approach on a table to document the identification, discrimination and localization of objects of daily living with white objects on a black background (non-reflective black tablecloth). A white fluorescent lamp equipped with a large diffusor positioned above the patients' heads yielded approximately 600–2000 lux at table level. The luminance of the objects was typically adjusted to 250–600 cd m^−2^, and the luminance of the black tablecloth was 5–15 cd m^−2^.

The subjects performed ADL with white geometrical shapes and tableware. On a black background, in the first part, four of six possible geometrical objects (square, circle, triangle, rectangle, ring or crescent) and in the second part, four of six tableware objects (small- and medium-sized plates, cup, fork, spoon and knife) were placed around a large white plate (figure 4*c,d*). The subjects were asked to report the number of observed objects (identification) in both set-ups, to locate them (localization) and to name them (discrimination). The performance scores for each question ranged from 0 to 4 (e.g. the identification score was 3 for three reported objects, and the discrimination score was 2 for two correctly named objects).

A Wilcoxon rank-test was performed for each question, visit and subject (*n* = 8). Scores in the ON and OFF power supply states were compared.

#### (iii) Letters

Spontaneous correct reading of Latin alphabet letters in contrast reversal (white on black background) was documented. The patient was not given any information regarding the letter choice. All the presented letters were visible within the visual field of the microchip or were smaller (5–10° visual field).

#### (iv) Patient-reported experiences in daily life

In the current clinical trial, the subjects were permitted to use the visual implant outdoors, at home, at their workplaces or on the street.

During first trial visit days, the mobility trainer accompanied the subjects during their visual experiences in daily life. Documentation of specific spontaneous perception was performed by videotaping the performance and experiences or by recording the patients' oral reports.

## 3. Results

In all nine enrolled subjects, the light-induced voltage changes generated by the implant that were assessed via electrical corneal recordings showed reliable and luminance-dependent signal generation.

The observation period of the subjects was three to nine months. In several patients, the observation period was limited by technical instability of the implant (for more information, see the electronic supplementary material, section ‘Stability of the implant system’) and was followed by the removal of the implant. In one case, a severe adverse event occurred. Subject S8 developed post-operative subretinal bleeding in the area of the implant, and the intraocular pressure increased to 46 mmHg. This issue was resolved with topical and general medication. This patient had a very good functional outcome. Details on the safety of the implant will be published separately after the trial.

In the first subject (S1), an intraoperative touch of the optic nerve head by the tip of the implant occurred and resulted in failure of light perception via the implant. This subject was therefore excluded from the results presented herein.

### (a) Standardized screen tasks

The remaining eight subjects (S2–S9) had light perception via the subretinal implant that was usually reported as a bright tilted square when looking at a homogeneously illuminated area. Light perception thresholds improved in the power ON compared with the power OFF state in all eight subjects (S2–S9). Seven subjects were able to localize a light wedge (figure 3*b*) on the screen, and five subjects were able to detect the motion of dot patterns (figure 3*b*) with an angular speed of up to 35 deg s^−1^. Grating acuity was successfully measured in six subjects, with a maximum of 3.3 cpd. Visual acuity was assessable with standardized Landolt C-rings in two subjects with the following results: 0.01 and 0.037 (decimal), corresponding to 20/2000 and 20/546 Snellen acuity or visual angles of 1.6° and 0.45°, or log(MAR) 2 and 1.43 respectively. The best function for each subject, as well as the set-up for the screen tasks, is presented in figure 3.

Light localization, motion detection, grating acuity and Landolt C-rings tasks failed to achieve significant response rates when the power supply was switched off.

### (b) Table tasks of activities of daily living

For all the following tasks, significant differences between the power ON and OFF scores were found: geometrical shape identification (*p* = 0.012), discrimination (*p* = 0.018) and localization (*p* = 0.012) and tableware object identification, discrimination and localization (*p* = 0.012 each), as illustrated in figure 4*a*,*b*.

### (c) Recognition of letters

Three subjects (S2, S6 and S8) were able to read at least several letters (e.g. T, V, L, I, O) spontaneously. Subject S4 needed some training to correctly discriminate among three letters in a three-alternative-forced-choice test. The patients reported seeing the letters as complete entities.

### (d) Patient reports of experiences in daily life

In the current implant, no colour vision is available. As all the microphotodiodes have equal spectral sensitivity, the electrodes address all three retinal cone pathways simultaneously. Five of the eight subjects (S2, S4, S5, S6 and S8) reported various implant-mediated visual perceptions in daily life [21]. In the near-vision range, the most relevant reports included the recognition of facial characteristics, such as mouth shapes (smiles) or the presence/absence of glasses, and differentiation between the contours of people and clothing patterns (striped patterns, black jacket versus white shirt). At home or at work, it was possible to visually localize or distinguish objects, such as telephones, cutlery, parts of the meal (light noodles versus dark beef), red wine versus white wine, and other objects, including door knobs, signs on doors, washbasins or wastebaskets.

In the far-vision range, the most frequently reported perception was finding the line of the horizon and objects along the horizon, such as houses or trees. A river was described as a bright, reflecting stripe. Cars on the street were localized on the basis of bright reflections from their surfaces; the same was true of glass windows in general. One patient reported recognizing stopping and moving cars at night due to their headlights, as well as recognition of the course of the street according to the alignment of the streetlights. Another patient reported seeing the contours of the heads of his colleagues during a work meeting. Other objects of appropriate contrast and size (within the 10°–15° visual field) were also recognized, such as a dark, square-shaped carpet in the next room, the stalk of a sunflower or a white goose swimming in a certain direction. One patient was able to read the letters of restaurant signs and store names.

Short movie sketches of some of the experiences are available via the electronic supplementary material, video S1.

### (e) Safety comment

The implantation surgery was successful in all cases with regards to placing the implant subretinally on the posterior eye pole. The intraoperative touch of the optic nerve in the first subject and one serious adverse event (subretinal post-operative bleeding with an increase of intraocular pressure up to 43 mmHg in S8, which was resolved without sequelae) present relevant complications of the technique in the presented group of patients. A detailed description of safety is discussed in a separate publication.

## 4. Discussion

This study showed that a subretinal implant can restore various visual functions in blind patients with hereditary retinal degeneration, as proved by standardized psychophysical laboratory testing, as well as by the subjects' own reports of their daily living activities and by observations of their performance indoors and outdoors.

So far, our approach using subretinal electronic implants is the only one that has successfully mediated images in a trial with freely moving blind persons by means of a light sensor array that moves with the eye. All the other current approaches require an extraocular camera that does not link image capture to eye movements, which, therefore, does not allow the utilization of microsaccades for refreshing the perceived images. Although the restoration of vision described here is limited, blind persons with no alternative therapy options regard this type of artificial vision as an improvement in everyday life.

### (a) Light and movement perception

Light perception via the implant was possible in all subjects except S1 (mentioned earlier) during the standardized tasks with full field stimulation. If full field light stimulation on the chip is applied, a tilted homogeneous white-yellowish square with an approximately 15° diagonal visual field is perceived. This field corresponds to the slightly tilted placing of the chip on the posterior pole of the eye. Parts of this visual area may be missing if retinal holes, insufficient vascularization of the overlying retina (retinal ischemia is a hallmark of hereditary retinal disease), or an intrinsic inability of the retina to process electrical signals owing to degenerative processes are present, as observed in subject S3. To overcome this difficulty as best we could, we developed a pre-operative planning procedure for optimal chip positioning on the fundus [16]. However, even with a smaller visual field, some fairly challenging tasks were performed correctly.

Image perception with the chip has a ‘blinking’ characteristic owing to the working frequency of the implant. In the majority of the subjects, this frequency was set to 5 Hz, meaning that image capture takes place five times per second with a 1 ms duration. However, some subjects preferred a higher frequency (up to 15 Hz) for more continuous perception. Two subjects could use frequencies of only 1–2 Hz because their images faded quickly at higher frequencies. This phenomenon may have been owing either to individual variances in neuronal refractory time during the electrical stimulation of a degenerated retina or to altered microsaccades that help to maintain stable perception in the healthy eyes.

Movement detection with the implant is limited not only by not fully restored retinal processing mechanisms but also by the working frequency of the device. Currently, the maximum recognizable speed is 35° per second, which is comparable to that of a car moving at 22 km h^−1^ at a distance of 10 m.

### (b) Visual acuity

Preclinical work [6] has shown that spatial resolution of electrical stimuli on the retina closer than 50 µm will be difficult to achieve. Assuming that 1° of visual angle corresponds to 280 µm of retinal distance [22,23], the density of the sensors (70 µm × 70 µm) is approximately four sensors per degree, resulting in a maximum decimal visual acuity of 0.06–0.08 (depending on the axial eye length). A visual acuity of 0.4 is needed for normal reading without visual aids, and a visual acuity of approximately 0.1 is needed for self-sustained orientation and navigation [24]. Values below 0.3 are considered ‘low vision’, and a visual acuity of less than 0.02 is defined as blindness according to German law. Therefore, our chip is technically able to transform blindness into low vision.

When we asked our subjects about the quality of the artificial vision, they described blurred images of the world in grey tones, which is reminiscent of unfocused images from an older black-and-white television set.

Grating acuity, in which the examinee is asked to identify the direction of stripes in a pattern (horizontal, etc.), is valuable for measuring spatial frequency resolution. In contrast to visual acuity, which is measured classically by optotypes such as letters, numbers or Landolt C-rings, grating acuity uses a larger field of view and is therefore measurable independently of foveal function or the recognition of optotypes and thus may provide the best general description of retinal resolution in artificial vision. A healthy human eye can resolve up to 30 grating periods within 1° of visual angle (30 cpd). Except for subjects S2 and S3, a grating acuity test was possible in all subjects in our trial, only two of whom achieved visual acuity with standard optotypes when using the implant (Landolt C-rings). S2 had a narrowed area of perception owing to a retinal hole, which may have led to failure in the grating acuity test. Instead of a periodic stripe pattern, she was able to differentiate a single stripe direction of 0.5° visual angle; however, owing to scatter, the image of the line on the retina may have been broader. S3 had generally weak perception via the implant and could use it only at a frequency of 1–2 Hz owing to fading of the image at higher frequencies.

The 3.3 cpd correctly recognized by subject S8 represents the limit of resolution that is possible with the subretinal chip currently in use. A conversion of 3.3 cpd to decimal visual acuity yields 0.1. We are treating this value with caution because it may be achievable only in special circumstances. The chip has four rows of electrodes per degree and, according to the Nyquist–Shannon sampling theorem, only half of this value, i.e. 2 cpd, can be resolved with certainty. Aliasing effects may arise if the spatial frequency of the grating is higher than that of the electrodes, causing perception of a lower-frequency pattern (Moiré effect), which then can be distinguished correctly.

The standard visual acuity as measured by Landolt C-rings in our trial was as high as 0.037 decimal (20/546) and was reproducible. Visual acuity with Landolt C-rings is derived from the visual angle of gap size in the ‘C’ ring. A visual acuity of 0.037 corresponds to a visual angle of 0.45° or a retinal distance of 126 µm, indicating that a segment of 1.8 subretinal sensors is sufficient for perception of the gap in the Landolt ring. Only three subjects succeeded in spontaneously reading letters. Surprisingly, this was not directly related to successful performance in the Landolt C-ring test, possibly because the gap in the Landolt ring is smaller than the gaps in regular letters.

### (c) Activities in daily life

Concerning the table tasks of activities in daily life, there was a significant improvement in visual function when the implant was activated. However, the degree of success differed from one subject to another. The detection of objects and their localization (‘how many?’ and ‘where?’) was more easily accomplished than shape recognition. Clearly, discrimination of objects requires a larger area of available visual field than localization and some useable spatial resolution. Subjects S3 and S7 practically failed in this task because both experienced pronounced fading, i.e. their implants allowed for non-fading perception at only 1 Hz within a reduced visual area.

An adjustment in chip sensitivity is possible across a wide range of luminance, from 1 to 100.000 cd m^−2^, which allows most patients to use the implant at night time and in dimly illuminated rooms, as well as outside on a bright sunny day. In the laboratory, for tests at the table, an object luminance of approximately 2000 cd m^−2^ was chosen according to the study plan. This relatively high value has three advantages: first, during screening for inclusion all patients undergo the same test to check the inclusion criterion ‘visual functions not appropriate for localization of objects, self-sustained navigation and orientation’ at high contrast levels; second, as high light levels can directly stimulate photosensitive melanopsin-containing ganglion cells, testing at high luminance levels excludes spatial perception in our subjects via such ganglion cells during screening; and third, some implants produce stronger perception and some produce weaker perception, depending on individual retinal states. At high-illumination levels with good contrast, the implants can be tested in comparable conditions, as chip sensitivity can easily be downregulated.

Five subjects reported useable visual experiences in daily life with the implant. They found this the most important and rewarding aspect. Only subjects S3 and S7 were unable to use the implant in daily life due to fading. S9 did not reach a subjectively relevant level of visual function in daily life, despite good results in the standardized tests.

### (d) Other current approaches in humans

Worldwide, several approaches to vision restoration via electronic implants are currently being tested [25–28]. Implants are under development that localize the electrical stimulation to subretinal [11,29–31], epiretinal [2] or suprachoroidal [3,4] space, or to cortical [32] or optic nerve [33] neurons. Not all of these devices have yet reached the clinical trial stage; currently, only the epiretinal Argus II (Second Sight Medical Products Inc.) prosthesis, which has received the CE mark (conforms with European Community legislative requirements such as safety) for use in Europe, and the subretinal alpha-IMS (Retina Implant AG) implant have been the subjects of long-term clinical studies.

The epiretinal prosthesis uses an external camera affixed to spectacles. It transfers a decoded electrical signal directly to the ganglion cells, the third visual pathway neurons, via 60 electrodes. This approach does not primarily involve the bipolar cells. Because the epiretinal array consists only of electrodes and has no light-sensitive elements, it does not require a clear cornea or lens. Accommodation and magnification are accomplished by zooming in the camera system, which can improve visual perception if focused on details [2]. Another advantage of the epiretinal approach is easier surgical insertion; the median implant surgery time is 4 h [2] compared with 6–8 h in the case of the subretinal implant. The epiretinal system has also shown long-term stability in the follow-ups of patients wearing the epiretinal prosthesis for up to 2.7 years [2]. By contrast, there are other advantages of subretinal implants. The epiretinal approaches bypass the neurons of the middle retinal layer, whereas subretinal implants make use of the natural processing power of the bipolar and amacrine cells that process a substantial amount of visual information, such as motion and contrast.

The use of an external camera eliminates the chance of using natural eye movements, which are important not only for visual search but also for preventing image fading on the retina through small involuntary eye movements that refresh images during visual perception. Thus, patients with an external camera may require continuous head movements to avoid image fading. Patients with subretinal light-sensitive implants, however, perceive the shapes of objects naturally from the first day of implantation onwards without reorganization of the spatial assignments of electrodes by an image processor and without scanning movements of the head. Our subretinal approach with autonomously functioning pixels allows a high electrode density of 1500 electrodes versus 60 electrodes in the epiretinal approach.

## 5. Summary

This monocentric study has shown that the wirelessly driven implant alpha-IMS, positioned in the subfoveolar subretinal space can restore useful vision in daily life for at least two-thirds of the blind patients investigated. The multicentre phase of the clinical study of the subretinal implant alpha-IMS (Retina Implant AG) has already begun at additional centres in Oxford, Hong Kong, London, Budapest and other centres. In particular, long-term stability and safety, as well as the development of visual recognition abilities via learning effects, over a longer observation period will be addressed in future patients who receive this implant.
